# THE UNITÉ RHUMATOLOGIQUE DES AFFECTIONS DE LA MAIN (URAM): TRANSLATION INTO PORTUGUESE, CULTURAL ADAPTATION AND VALIDATION

**DOI:** 10.1590/1413-785220263403e299657

**Published:** 2026-06-22

**Authors:** Rodrigo Guerra Sabongi, Yan Celuppi Dal Vesco, Luiz Guilherme de Saboya Lenzi, João Baptista Gomes dos Santos, João Carlos Belloti, Flávio Faloppa

**Affiliations:** 1 Universidade Federal de Sao Paulo (UNIFESP), Escola Paulista de Medicina, Departamento de Ortopedia e Traumatologia, Sao Paulo, SP, Brazil.

**Keywords:** Dupuytren Contracture, Surveys and Questionnaires, Validation Study, Hand, Translating, Contratura de Dupuytren, Inquéritos e Questionários, Estudo de Validação, Mãos, Tradução

## Abstract

**Objective:**

To translate and culturally adapt the Unité Rhumatologique des Affections de la Main (URAM) scale, a specific instrument for functional assessment in Dupuytren's disease (DD), into Brazilian Portuguese.

**Methods:**

Following the methodology of Beaton (2000), the scale underwent five stages: translation, synthesis, back-translation, expert committee, and pre-testing. After adaptation, the Brazilian version of the scale (URAM-BR) was administered to 40 patients with Dupuytren's contracture. Internal consistency was tested using Cronbach's alpha, reproducibility was analyzed using the intraclass correlation coefficient (ICC), and construct validity was analyzed by correlation with the Brazilian version of QuickDASH.

**Results:**

The URAM-BR showed excellent internal consistency (α = 0.930–0.938), significant reproducibility (ICC > 0.89), and moderate-to-strong construct validity (r = 0.69, p < 0.001). The adapted version proved to be clear, reliable, and appropriate to the Brazilian cultural context.

**Conclusion:**

The URAM-BR is a valid, reliable, and reproducible instrument for functional assessment of patients with DD in Brazil. ***Level of evidence IV; Observational cross-sectional instrument validation study.***

## RESUMO

**Objetivo:**

Traduzir e adaptar culturalmente para o português do Brasil a escala Unité Rhumatologique des Affections de la Main (URAM), instrumento específico para avaliação funcional na Doença de Dupuytren (DD).

**Métodos:**

Seguindo a metodologia de Beaton (2000), a escala passou por cinco etapas: tradução, síntese, retrotradução, comitê de especialistas e pré-teste. Após a adaptação, a versão brasileira da escala (URAM-BR) foi aplicada em 40 pacientes com contratura de Dupuytren. A consistência interna foi testada pelo alfa de Cronbach, a reprodutibilidade foi analisada pelo Coeficiente de Correlação Intraclasse (ICC), e a validade de construto pela correlação com a versão brasileira da QuickDASH.

**Resultados:**

O URAM-BR apresentou excelente consistência interna (α = 0,930–0,938), reprodutibilidade significativa (ICC > 0,89) e validade de construto moderada-forte (r = 0,69, p < 0,001). A versão adaptada demonstrou ser clara, confiável e adequada ao contexto cultural brasileiro.

**Conclusão:**

O URAM-BR é um instrumento válido, confiável e reprodutível para avaliação funcional de pacientes com DD no Brasil. ***Nível de evidência IV; estudo observacional transversal de validação de instrumento.***

## INTRODUCTION

Dupuytren's disease (DD) is characterized by the proliferation and thickening of the palmar fascia, which occurs through the differentiation of fibroblasts into myofibroblasts, cells responsible for the excessive production of collagen^1^, forming nodules that can evolve into rigid cords, with collagen fibers organized parallel to the lines of tension. About 10% of patients with DD progress to progressive, permanent flexion of the affected fingers, most commonly the ring and little fingers, which characterizes Dupuytren's contracture (DC)^2^. Flexion contractures affect the metacarpophalangeal (MCP) joints, proximal interphalangeal (PIP) joints, and, more rarely, the distal interphalangeal (DIP) joints, which can lead to severe deformities^3^. The inability to fully open the fingers will affect patients’ ability to handle objects, which consequently leads to reports of limitations in work, leisure activities, and self-care, such as using a computer, playing instruments, practicing sports, and performing personal hygiene^4^.

Assessing the patient's perception of their limitations is important to clarify and tailor Dupuytren's disease treatment to individual needs and expectations, which can be done using patient-reported outcome measures (PROMs). PROMs questionnaires specific to the affected segment, such as the *Disabilities of the Arm, Shoulder and Hand* (DASH), the *Michigan Hand Questionnaire* (MHQ), and their shortened versions, QuickDASH and the *Brief Michigan Hand Questionnaire* (BMHQ), have been translated into Brazilian Portuguese^5–7^ and are widely used in the evaluation of patients and outcomes in clinical studies on various hand-affecting pathologies. However, they are nonspecific to the limitations presented by patients with Dupuytren's disease and are not very sensitive to changes in the range of motion that occur with disease progression and treatment of contractures^8^.

The *Unité Rhumatologique des Affections de la Main* (URAM) was the first PROMs developed and validated specifically for Dupuytren's disease, consisting of 9 questions that assess the patient's difficulty in performing some daily living activities commonly affected by finger extension limitations. Its scoring ranges from 0 to 5 for each item, totaling up to 45 points, where higher values represent greater functional limitation^9^. The URAM has validated versions in English, French, Italian, Spanish, German, Dutch, and Korean^10–14^, but has not yet been translated into Portuguese.

We developed this study to translate the URAM questionnaire into Portuguese and adapt it to our country's culture for use in the Brazilian population with Dupuytren's disease.

## MATERIALS AND METHODS

### Research Design

This study is a cross-sectional, observational, primary research study focused on validating a questionnaire for assessing Dupuytren's disease.

Adult patients diagnosed with Dupuytren's contracture were included in the present sample, while those under 18 years of age or who did not agree to sign the Informed Consent Form (ICF) were excluded. The project was approved by the Research Ethics Committee (REC), under opinion number 7.510.123, CAAE: 85528624.1.0000.5505. The translation of the URAM questionnaire was carried out according to the translation, cultural adaptation, and validation protocol proposed by Beaton et al. (2000), which comprises five stages: translation, synthesis, back-translation, expert committee, and pre-testing.^15^

### Translation and Cultural Validation Stages

#### Translation

Two translators fluent in French, with Portuguese as their native language, independently translated the URAM questionnaire from the original French version. One of them was a hand surgery specialist with experience in the clinical management of Dupuytren's disease, while the other was a sworn translator with no prior knowledge of the pathology. The resulting translations were designated T1 and T2, respectively. (see Appendix 1)

#### Synthesis

The translators gathered to analyze the discrepancies between versions T1 and T2 and synthesize them into a consensual translation. The main divergence was observed in item 1, which originally referred to the term "*toilet bag*", a common object in personal hygiene habits in European countries, but little used by the Brazilian population. Understanding that the question aimed to describe a hygiene action during bathing that requires the use of an open hand to hold an object in contact with the body, it was decided to culturally adapt it to "can you lather up with your open hand?". With this modification, and after consensus among the translators, the unified version of the translation, called T12, was generated. (see Appendix 1)

#### Back Translation

To verify the consistency between the original French version and version T12, two independent translators, whose native language is French, fluent in Portuguese, not belonging to the medical field, and blind to the original questionnaire, performed the back translation of T12 into French, generating versions RT1 and RT2. Except for item 1, which was modified for cultural adaptation as previously described, all other questions presented semantic equivalence with the original version of the URAM. (see Appendix 1)

#### Expert Committee

The expert committee was composed of the translators, one hand surgery resident, and three hand surgeons, with different levels of professional experience: one with less than 10 years, two with more than 10 years, and one with over 30 years in the field. The group met to evaluate consistency between the translated and back-translated versions, equivalence relative to the original version, and the cultural appropriateness of the items. Based on this analysis, the version of the questionnaire to be applied to patients was defined.

#### Pre-Test

The version of the questionnaire obtained after the expert committee was applied to 40 patients who attended for outpatient evaluation at the institution and were diagnosed with DC, all of whom had previously agreed to participate by signing the ICF.

Demographic data were collected, including sex and age, degree of contracture of the affected rays using a goniometer, time required to complete the questionnaire, and identification of unanswered or incorrectly filled items. The degree of patients’ understanding of each item was also assessed, and any doubts were recorded. Based on this preliminary stage, we reviewed the translated version and finalized the instrument in Brazilian Portuguese, called URAM-BR.

### Statistical Analysis

The collected data were organized in Microsoft Excel spreadsheets and analyzed using IBM SPSS Statistics software (IBM Corp. Released 2023. IBM SPSS Statistics for Windows, Version 29.0.2.0. Armonk, NY: IBM Corp). For all analyses, a significance level of 5% (p < 0.05) was adopted.

The reproducibility of the URAM questionnaire was assessed through interviews with patients, by comparing responses obtained in the pre-test and retest, using the Intraclass Correlation Coefficient (ICC), which measures the stability of responses over time, as well as the observed integrity.

The internal reliability of the questionnaire was assessed using Cronbach's alpha, which evaluates the internal consistency of responses across items for different patients. Additionally, a construct validity analysis was conducted, comparing the URAM scores with those of the QuickDASH scale, which has already been validated for functional assessment in Dupuytren's disease, to assess convergence between the instruments and the ability of the new questionnaire to produce equivalent results.

## RESULTS

The clinical and demographic characteristics of the patients are presented in Table 1. The average age was 64 years. Of the 40 participants, 32 (80%) were male, 37 (92.5%) were right-handed, and 29 (72.5%) had bilateral involvement of Dupuytren's disease. All patients reported at least one comorbidity, and the majority (n = 26; 65%) were in postoperative follow-up.

**Table 1:** Patient characteristics.

Characteristics	n (%) mean (SD)
Age	64.93 (6.45)
**Sex**	
Male	32 (80%)
Female	8 (20%)
**Dominant side**	
Right-handed	37 (92.5%)
Left-handed	3 (7.5%)
**Comorbidity count**	
1	23 (57.5%)
2	8 (20%)
3	5 (12.5%)
4	3 (7.5%)
5	1 (2.5%)
**Hands affected by Dupuytren**	
Right	8 (20%)
Left	3 (7.5%)
Bilateral	29 (72.5%)
**Disease status**	
Pre-operative	10 (25%)
Conservative	4 (10%)
Post-operative	26 (65%)
URAM Sum (1st application)	20.78 (12.85)
URAM Sum (2nd application)	20.83 (12.77)
QuickDASH Score	41.02 (23.05)

Values indicated in count (% of the group) or average (standard deviation). as appropriate. N=40

**Figure 1:**
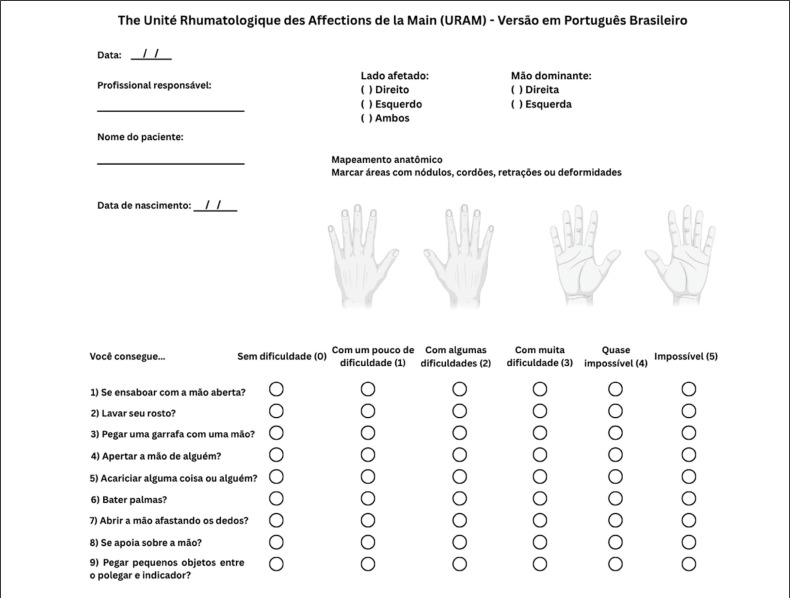
URAM-BR, URAM's brazilian versions (*Unité Rhumatologique des Affections de la Main*).

Table 2 presents patients’ evaluation of the clarity of the questions. At least 92.5% of participants considered each questionnaire item clear. The greatest difficulty was observed in question 7, regarding the movement of opening the hand by spreading the fingers, which three patients (7.5%) marked as unclear. On the other hand, question 6, "clap your hands?", was the only one considered clear by all 40 participants, demonstrating total understanding. The scale in Portuguese is presented in Figure 1.

**Table 2:** Evaluation of the questions by the patients.

Item	n (%)
**1) Do you wash with an open hand?**	
Clear	39 (97.5)
Not clear	1 (2.5)
**2) Do you wash your face?**	
Clear	39 (97.5)
Not clear	1 (2.5)
**3) Can you pick up a bottle with one hand?**	
Clear	39 (97.5)
Not clear	1 (2.5)
**4) Can you shake someone's hand?**	
Clear	39 (97.5)
Not clear	1 (2.5)
**5) Can you pet something or someone?**	
Clear	38 (95)
Not clear	2 (5)
**6) Can you clap your hands?**	
Clear	40 (100)
Not clear	0 (0)
**7) Can you open your hand by spreading your fingers?**	
Clear	37 (92.5)
Not clear	3 (7.5)
**8) Can you lean on your hand?**	
Clear	39 (97.5)
Not clear	1 (2.5)
**9) Can you pick up small objects between your thumb and index finger?**	
Clear	38 (95)
Not clear	2 (5)

Values indicated in count (% of the group). N=40.

The analysis of internal consistency, presented in Table 3, revealed a Cronbach's alpha coefficient of 0.930 in the first application of the test, indicating excellent internal reliability of the instrument.

**Table 3:** Internal consistency of URAM responses in the first test.

Item	Mean (SD)	Alfa de Cronbach	
		When excluding the item*	General
1) Do you wash with an open hand?	2.42 (1.796)	0.920	0.930
2) Do you wash your face?	2.42 (1.852)	0.919	
3) Can you pick up a bottle with one hand?	2.00 (1.649)	0.919	
4) Can you shake someone's hand?	2.20 (1.757)	0.923	
5) Can you pet something or someone?	2.00 (1.769)	0.926	
6) Can you clap your hands?	2.85 (1.875)	0.919	
7) Can you open your hand by spreading your fingers?	3.05 (1.880)	0.923	
8) Can you lean on your hand?	3.00 (1.974)	0.929	
9) Can you pick up small objects between your thumb and index finger?	0.83 (1.299)	0.942	

Mean and standard deviation of the responses to each of the questions on the *URAM* scale. The scale ranges from 1 to 5. N=40. *Describes the adjusted Cronbach's Alpha coefficient if the respective item is excluded from the construct.

In the second application of the URAM, the analysis of internal consistency yielded a Cronbach's alpha coefficient of 0.938, reinforcing the instrument's high reliability (Table 4).

**Table 4:** Internal consistency of URAM responses (second test).

Item	Mean (SD)	Alfa de Cronbach	
		When excluding the item*	General
1) Do you wash with an open hand?	2.42 (1.767)	0.929	0.938
2) Do you wash your face?	2.40 (1.851)	0.928	
3) Can you pick up a bottle with one hand?	2.08 (1.623)	0.929	
4) Can you shake someone's hand?	2.20 (1.667)	0.929	
5) Can you pet something or someone?	2.03 (1.747)	0.935	
6) Can you clap your hands?	2.78 (1.819)	0.929	
7) Can you open your hand by spreading your fingers?	3.10 (1.823)	0.932	
8) Can you lean on your hand?	3.03 (1.874)	0.940	
9) Can you pick up small objects between your thumb and index finger?	0.80 (1.224)	0.953	

Mean and standard deviation of the responses to each of the questions on the *URAM* scale. The scale ranges from 1 to 5. N=40. *Describes the adjusted Cronbach's Alpha coefficient if the respective item is excluded from the construct.

Table 5 presents the reproducibility analysis, with the ICC calculated for each questionnaire item. All values were equal to or greater than 0.897, indicating at least good reproducibility for all items. Most questions showed an ICC above 0.90, indicating excellent reproducibility and supporting the stability of responses between the two applications of the URAM-BR.

**Table 5:** Reproducibility analysis.

Item	Mean (SD)		
	1st test	2nd test	ICC
1) Do you wash with an open hand?	2.42 (1.796)	2.42 (1.767)	0.968
2) Do you wash your face?	2.42 (1.852)	2.40 (1.851)	0.960
3) Can you pick up a bottle with one hand?	2.00 (1.649)	2.08 (1.623)	0.958
4) Can you shake someone's hand?	2.20 (1.757)	2.20 (1.667)	0.897
5) Can you pet something or someone?	2.00 (1.769)	2.03 (1.747)	0.955
6) Can you clap your hands?	2.85 (1.875)	2.78 (1.819)	0.952
7) Can you open your hand by spreading your fingers?	3.05 (1.880)	3.10 (1.823)	0.956
8) Can you lean on your hand?	3.00 (1.974)	3.03 (1.874)	0.956
9) Can you pick up small objects between your thumb and index finger?	0.83 (1.299)	0.80 (1.224)	0.945

Reproducibility analysis of the URAM test by *Intraclass Correlation Coefficients* - ICC) intra-observer. Results of the questions expressed as mean and standard deviation for the 1st and 2nd application of the test. The parameters for the ICC test were established, the mixed model of two factors, with single measures.

Table 6 presents the correlation analysis of the URAM and QuickDASH scores. A statistically significant correlation was observed in both applications of the URAM (p < 0.001 in both cases). The Spearman correlation coefficients were 0.690 in the first application and 0.695 in the second, indicating a moderate-to-strong correlation. QuickDASH was applied only once.

**Table 6:** Construct analysis, comparative between URAM and QuickDASH.

Item	Mean (SD)	p	R
URAM Sum (1st application)	20.78 (12.85)	<0.001	0.690
URAM Sum (2nd application)	20.83 (12.77)	<0.001	0.695
QuickDASH Score	41.02 (23.05)	-	1.000

Assessment of the construct of URAM by correlation with the QuickDASH scale. Results indicated as mean (standard deviation). N=40. *r* = Spearman's coefficient.

## DISCUSSION

The use of appropriate instruments to assess patients with Dupuytren's disease is essential for systematically documenting disease progression and treatment outcomes. In this context, patient-reported outcome measures (PROMs) provide a more sensitive and patient-centered perspective on the functional impact of the disease, overcoming the limitations of isolated objective measures, such as the degree of contracture in specific joints^16^.

The literature has pointed out the lack of standardization among the instruments used in clinical studies with patients with Dupuytren's disease, which compromises the comparability of findings and hinders the consolidation of scientific evidence^17^. The cultural adaptation and validation of already established instruments, such as the URAM, following rigorous protocols like those of Beaton et al.^15^, ensure that their psychometric properties are preserved in new linguistic and cultural contexts.

The URAM is, to date, the only PROM developed specifically to assess functionality in patients with Dupuytren's disease. Previous studies have validated this instrument in languages such as English, German, Dutch, Korean, Spanish, and Italian^9–14^. The present study was the first to adapt the URAM for Brazilian Portuguese, with results that attest to its conceptual, cultural, and statistical equivalence to the original version.

Figure 1 presents the final version of the URAM-BR, developed in simple language and with a standardized structure, facilitating its clinical application. The instrument comprises nine items related to daily manual activities, scored from 0 (no difficulty) to 5 (impossible), according to the degree of perceived functional limitation. Auxiliary anatomical representations for mapping nodules and retractions reinforce the practical and patient-centered nature of the adapted version.

The Brazilian version of the URAM demonstrated excellent internal consistency, with Cronbach's alpha coefficients of 0.930 and 0.938. According to Landis and Koch^18^, this coefficient indicates very high consistency among the construct's responses. Such values indicate high homogeneity among the items and suggest that the questions coherently assess the same construct: functional limitation related to Dupuytren's disease. These findings are comparable to those of international studies: the Italian version presented a Cronbach's alpha coefficient of 0.94^10^, the Spanish version 0.85^11^, while the German^12^, Dutch^13^, and Korean^14^ versions presented an alpha of 0.91, corroborating the stability of the instrument across different cultures.

The reproducibility of the URAM-BR was also excellent. The intraclass correlation coefficients ranged from 0.897 to 0.968 across the questions, confirming the stability of responses over time. According to the criteria of Koo and Li^19^, ICC values above 0.90 are considered indicative of excellent reproducibility, while values between 0.75 and 0.90 are good. These results support the use of URAM-BR in clinical and research contexts that require stable responses over time. The reproducibility demonstrated in this study was superior to that of the Dutch version, which presented an ICC of 0.76 (0.64-0.87)^13^, and was comparable to the Korean version, with an ICC of 0.89 (0.78-0.95)^14^, to the Spanish version, with an ICC of 0.93 (0.88–0.96)^11^, and to the Italian version, with an ICC of 0.96 (0.94–0.98)^10^. The German version^12^ did not present ICC values for comparison.

Construct validity was evidenced by a statistically significant correlation with the Brazilian version of the QuickDASH scale, a widely used instrument that is not specific to Dupuytren's disease (DD)^8^. The Spearman correlation coefficients were 0.690 and 0.695 for the first and second applications of the URAM, respectively, both statistically significant (p < 0.001). According to Cohen's classification, these values indicate a moderate-to-strong correlation, demonstrating that URAM-BR comparably measures the functional aspects of the upper limb, with the advantage of specifically capturing the limitations imposed by the progressive digital contracture of DD^20^. The Spanish version also used QuickDASH as a comparative instrument and found a slightly higher Spearman correlation coefficient, r = 0.716^11^. In contrast, the Korean and Italian versions employed Pearson correlation with the PRWHE-I and K-PRWE, presenting values of r = 0.60 ^(10)^ and r = 0.56 ^(14)^, respectively. The German validation demonstrated a higher inverse correlation between URAM and the reduced total score of the MHQ, r = −0.76^12^, while the Dutch version found r = −0.65 in comparison with the MHQ^13^.

The qualitative analysis of item comprehension indicated a high clarity rate in all items (≥ 92.5%). This high understanding rate attests to the effectiveness of the cultural adaptation process, especially in modifying item 1, which originally referred to the use of *gant de toilette*, an object that is not common in Brazil, and was adapted to washing with an open hand, respecting functional and semantic equivalence^15^.

Despite the promising results, this study has relevant limitations. Firstly, the sample size was small (n = 40), which limits the generalizability of the findings. Additionally, there was a predominance of postoperative patients (65%), limiting comparisons across clinical stages of the disease, such as the pre-treatment and conservative follow-up phases. No sensitivity-to-change analysis (responsiveness) was conducted, an essential criterion for the instrument's use in longitudinal studies of therapeutic efficacy. Finally, the application of URAM-BR has not yet been tested in different regional and socioeconomic contexts in Brazil, which would be fundamental to ensure its external validity and national applicability.

Ultimately, URAM-BR emerges as a valuable tool for both clinical practice and scientific research in hand rehabilitation and surgery. Its brief format, accessible language, clarity in questions, and ability to capture the specific functional limitations of DD make it ideal for routine application. The availability of a validated version in Brazilian Portuguese fills an important gap in the functional assessment of DD.

## CONCLUSION

The URAM questionnaire was translated and culturally adapted, and its psychometric properties were tested in the Brazilian population, resulting in the URAM-BR, a clear, easy-to-use instrument with excellent internal consistency, reproducibility, and construct validity.

## Data Availability

Data will be made available upon request.

## Appendix 1

**Table:**

T1:						
Can you…	Without difficulty (0)	With a little difficulty (1)	With some difficulties (2)	With a lot of difficulty (3)	Almost impossible (4)	Impossible (5)
1) Wash yourself with a hand towel?	▢	▢	▢	▢	▢	▢
2) Wash your face?	▢	▢	▢	▢	▢	▢
3) Pick up a bottle with one hand?	▢	▢	▢	▢	▢	▢
4) Shake someone's hand?	▢	▢	▢	▢	▢	▢
5) Pet something or someone?	▢	▢	▢	▢	▢	▢
6) Clap your hands?	▢	▢	▢	▢	▢	▢
7) Completely separate your fingers?	▢	▢	▢	▢	▢	▢
8) Support oneself with one hand?	▢	▢	▢	▢	▢	▢
9) Pick up small objects between the thumb and index finger?	▢	▢	▢	▢	▢	▢

**Table:**

T2:						
Can you…	Without difficulty (0)	With very little difficulty (1)	With some difficulties (2)	With a lot of difficulty (3)	Almost impossible (4)	Impossible (5)
1) Wash oneself with a bath glove?	▢	▢	▢	▢	▢	▢
2) Wash the face?	▢	▢	▢	▢	▢	▢
3) Pick up a bottle with one hand?	▢	▢	▢	▢	▢	▢
4) Shake someone's hand?	▢	▢	▢	▢	▢	▢
5) Caress anything or anyone?	▢	▢	▢	▢	▢	▢
6) Clap?	▢	▢	▢	▢	▢	▢
7) Spread the fingers apart?	▢	▢	▢	▢	▢	▢
8) Lean on the hand?	▢	▢	▢	▢	▢	▢
9) Grab small objects between the thumb and index finger?	▢	▢	▢	▢	▢	▢

**Table:**

T12:						
Can you…	Without difficulty (0)	With a little difficulty (1)	With some difficulties (2)	With a lot of difficulty (3)	Almost impossible (4)	Impossible (5)
1) Lather oneself with an open hand?	▢	▢	▢	▢	▢	▢
2) Wash your face?	▢	▢	▢	▢	▢	▢
3) Pick up a bottle with one hand?	▢	▢	▢	▢	▢	▢
4) Shake someone's hand?	▢	▢	▢	▢	▢	▢
5) Pet something or someone?	▢	▢	▢	▢	▢	▢
6) Clap your hands?	▢	▢	▢	▢	▢	▢
7) Open the hand by spreading the fingers apart?	▢	▢	▢	▢	▢	▢
8) Lean on the hand?	▢	▢	▢	▢	▢	▢
9) Pick up small objects between the thumb and index finger?	▢	▢	▢	▢	▢	▢

**Table:**

RT1:						
Pouvez-vous…	Sans difficulté (0)	Avec un peu de difficulté (1)	Avec une certaine difficulté (2)	Avec beaucoup de difficulté (3)	Presque impossible (4)	Impossible (5)
1) Vous savonner avec la main ouverte?	▢	▢	▢	▢	▢	▢
2) Vous laver le visage?	▢	▢	▢	▢	▢	▢
3) Prendre une bouteille d'une main?	▢	▢	▢	▢	▢	▢
4) Serrer la main de quelqu'un?	▢	▢	▢	▢	▢	▢
5) Caresser quelque chose ou quelqu'un?	▢	▢	▢	▢	▢	▢
6) Applaudir?	▢	▢	▢	▢	▢	▢
7) Ouvrir la main en écartant les doigts?	▢	▢	▢	▢	▢	▢
8) Prendre appui sur la main?	▢	▢	▢	▢	▢	▢
9) Prendre de petits objets entre le pouce et l'index?	▢	▢	▢	▢	▢	▢

**Table:**

RT2:						
Pouvez-vous…	Sans difficulté (0)	Avec un peu de difficultés (1)	Avec une certaine difficultés (2)	Avec beaucoup de difficultés (3)	Presque impossible (4)	Impossible (5)
1) Vous savonner avec la main ouverte?	▢	▢	▢	▢	▢	▢
2) Laver votre visage?	▢	▢	▢	▢	▢	▢
3) Prendre une bouteille avec la main?	▢	▢	▢	▢	▢	▢
4) Serrer la main de quelqu'un?	▢	▢	▢	▢	▢	▢
5) Caresser quelque chose ou quelqu'un?	▢	▢	▢	▢	▢	▢
6) Applaudir?	▢	▢	▢	▢	▢	▢
7) écarter les doigts les uns des autres?	▢	▢	▢	▢	▢	▢
8) Vous appuyer sur la main?	▢	▢	▢	▢	▢	▢
9) Prendre de petits objets entre le pouce et l'index?	▢	▢	▢	▢	▢	▢
